# Emergence of *mcr-1*-Harboring *Salmonella enterica* Serovar Sinstorf Type ST155 Isolated From Patients With Diarrhea in Jiangsu, China

**DOI:** 10.3389/fmicb.2021.723697

**Published:** 2021-09-03

**Authors:** Guoye Liu, Huimin Qian, Jingwen Lv, Benshun Tian, Changjun Bao, Hong Yan, Bing Gu

**Affiliations:** ^1^Department of Clinical Laboratory, the Affiliated Drum Tower Hospital of Nanjing University Medical School, Nanjing, China; ^2^Department of Acute Infectious Disease Prevention and Control, Jiangsu Provincial Center for Disease Prevention and Control, Nanjing, China; ^3^Laboratory Medicine, Guangdong Provincial People’s Hospital, Guangdong Academy of Medical Sciences, Guangzhou, China; ^4^Laboratory Medicine Center, The Second Affiliated Hospital, Nanjing Medical University, Nanjing, China

**Keywords:** plasmid-borne quinolone resistance, extended-spectrum beta-lactamases, quinolone resistance determining region, nontyphoidal *Salmonella*, *mcr-1*

## Abstract

**Background:** This study analyzed the antimicrobial resistance phenotypes and mechanisms of quinolone, cephalosporins, and colistin resistance in nontyphoidal *Salmonella* from patients with diarrhea in Jiangsu, China.

**Methods:** A total of 741 nontyphoidal *Salmonella* isolates were collected from hospitals in major cities of Jiangsu Province, China between 2016 and 2017. Their susceptibility to commonly used antibiotics was evaluated by broth micro-dilution and sequencing analysis of resistance genes screened by a PCR method. For *mcr-1* positive isolates, genetic relationship study was carried out by pulsed-field gel electrophoresis and multiloci sequence typing analysis. The transferability of these plasmids was measured with conjugation experiments and the genetic locations of *mcr-1* were analyzed by pulsed-field gel electrophoresis profiles of S1-digested genomic DNA and subsequent Southern blot hybridization.

**Results:** Among 741 nontyphoidal *Salmonella* isolates, the most common serotypes identified were *S.* Typhimurium (*n*=257, 34.7%) and *S.* Enteritidis (*n*=127, 17.1%), and the isolates showed 21.7, 20.6, and 5.0% resistance to cephalosporins, ciprofloxacin, and colistin, respectively. Among the 335 nalidixic acid-resistant *Salmonella*, 213 (63.6%) and 45 (13.4%) had at least one mutation in *gyrA* and *parC*. Among the plasmid-borne resistance, *qnrS1* (85; 41.9%) and *aac(6')-Ib-cr4* (75; 36.9%) were the most common quinolone resistance (PMQR) genes, while *bla*_CTX-M-14_ (*n*=35) and *bla*_CTX-M-55_ (*n*=46) were found to be dominant extended-spectrum beta-lactamase (ESBL) genes in nontyphoidal *Salmonella*. In addition, eight *mcr-1*-harboring strains were detected since 2016 and they were predominate in children under the age of 7years. Conjugation assays showed the donor *Salmonella* strain has functional and transferable colistin resistance and Southern blot hybridization revealed that *mcr-1* was located in a high molecular weight plasmid.

**Conclusion:** In nontyphoidal *Salmonella*, there is a rapidly increasing trend of colistin resistance and this is the first report of patients harboring *mcr-1*-positive *Salmonella* with a new ST type ST155 and new serotype *S.* Sinstorf. These findings demonstrate the necessity for cautious use and the continuous monitoring of colistin in clinical applications.

## Introduction

Antibiotic resistance is an ongoing severe threat to global health, food security, and development today, and it may affect anyone, of any age, in any country. Antibiotic resistance is rising to dangerously high levels in all parts of the world. According to WHO data, the median rate observed for methicillin-resistant *Staphylococcus aureus* was 12.11% in 25 countries and that for *Escherichia coli* resistant to third-generation cephalosporins was 36.0% in 49 countries in 2019. How and why is this happening (Lin et al., 2015)? Related reports have found that the mechanisms include: (1) Bacteria restrict access by antibiotics by changing their entry ways or limiting the number of entry ways. (2) Bacteria get rid of antibiotics by using pumps in their cell walls that remove any antibiotic drugs that enter the cell. (3) Bacteria change or destroy the antibiotics with enzymes that break down the drug. (4) Bacteria develop new cell processes that bypass the effects of the antibiotic. (5) Bacteria change the antibiotic’s target so the drug can no longer recognize it and do its job. In addition, new resistance mechanisms keep emerging and spreading globally, threatening our ability to treat common infectious diseases. A growing list of infections such as pneumonia, tuberculosis, blood poisoning, gonorrhea, and foodborne diseases are becoming harder, and sometimes impossible, to treat as antibiotics become less effective, leading to higher medical costs, prolonged hospital stays, and increased mortality (Elkington, 2020).

Nontyphoidal *Salmonellae* refers to illnesses caused by all serotypes of *Salmonella* except for *Salmonella enterica* serovar Typhi and paratyphoidal serovars (i.e., serovars Paratyphi A, Paratyphi B, and Paratyphi C). They are leading causes of bacterial diarrhea worldwide. *Salmonella* is a Gram-negative rods genus belonging to the *Enterobacteriaceae* family. They are frequently transmitted from food or water contaminated with animal feces, or infection occurs through direct contact with infected animals or their environment and also directly between humans, causing numerous gastroenteritis cases. In addition to diarrheal disease, nontyphoidal *Salmonella* infections can invade normally sterile sites, resulting in bacteremia, meningitis, and other focal infections. The Global Burden of Diseases (GBD), Injuries, and Risk Factors Study 2017 estimated that *Salmonella* enterocolitis caused about 95 million infections, 50,000 deaths, and 3 million disability adjusted life-years in 2017 (GBD 2017 Causes of Death Collaborators, 2018; GBD 2017 DALYs and HALE Collaborators, 2018; GBD 2017 Disease and Injury Incidence and Prevalence Collaborators, 2018). Travelling is the main risk factor for nontyphoidal *Salmonella* infection, and it is estimated that among travelers returning to the United States, the highest risk is those who visited Africa (25.8 cases per 100,000 air travelers). In addition, *Salmonella* infection and carriage has been reported among internationally adopted children.

Antimicrobial resistance in nontyphoidal *Salmonella* serotypes has become a global problem. With the extensive use of antimicrobials, antimicrobial resistance is increasing at a serious rate in *Salmonella* isolates. In many countries, increasing numbers of *Salmonella* strains with MDR (defined as resistance to three or more classes of antimicrobials) have been discovered since the report of the spread of MDR in *S.* Typhimurium of definitive phage type 104 (DT104) around the world (Molbak et al., 1999). Since then, nontyphoidal *Salmonella* in Europe, Southeast Asia and other places have also developed resistance to the ciprofloxacin-fluoroquinolone drugs. Mutations in the quinolone resistance determining region (QRDR) may reduce their sensitivity to fluoroquinolone drugs, which depends on the number of mutations obtained. Third-generation cephalosporin drugs are used as a treatment for salmonellosis in areas with high levels of quinolone resistance because of its good effect on enteritis. However, ESBL or AmpC type β-lactamase mediated resistance to extended-spectrum cephalosporins of *Salmonella* has emerged. Additionally, in 2016, a plasmid encoding *mcr-1*-mediated colistin resistance in *Enterobacteriaceae* was newly recognized. Its existence presents a severe threat in the global response to antibiotic resistance (Liu et al., 2016). Furthermore, MDR *Salmonella*, which are important agents in the transmission of antibiotic resistance genes, have become a severe medical threat (Schrijver et al., 2018).

In this study, we analyzed the antimicrobial resistance profiles of nontyphoidal *Salmonella* in Jiangsu from 2016 to 2017 and elucidated the molecular mechanisms underlying the emergence of MDR in these isolates. Our findings will help provide appropriate clinical antimicrobial treatment for patients with nontyphoidal *Salmonella* infection in Jiangsu of eastern China.

## Materials and Methods

### Specimen Collection and Isolate Identification

A total of 741 fresh stool samples from clinically suspected patients who had diarrhea were collected from different hospitals located in 13 cities of Jiangsu Province between 2016 and 2017. The stool samples were then cultivated on *Salmonella-Shigella* (SS) xylose lysine deoxycholate (XLD) agar (XLD; CHRO Magar, China) and incubated for 18–24h at 37°C. API20 Etest strips (bioMerieux Vitek, Marcy-l’Etoile, France) were used to confirm the identity of the isolates. All of the isolates were then serotyped by slide agglutination with commercial antiserum (Tianrun Bio-Pharmaceutical Co., Ltd., China) according to the Kauffmann-White Scheme (World Health Organization, 2011).

### Antimicrobial Susceptibility Testing

Antimicrobial susceptibility testing was performed on the nontyphoidal *Salmonella* isolates for 30 antimicrobial agents: ampicillin (AMP), ampicillin/sulbactam (AMS), tetracycline (TET), chloramphenicol (CHL), trimethoprim/sulfamethoxazole (SXT), cefazolin (FAZ), cefotaxime (CTX), ceftazidime (CAZ), cefoxitin (FOX), gentamicin (GEN), imipenem (IMI), nalidixic acid (NAL), azithromycin (AZI), sulfa isoxazole (Sul), ciprofloxacin (CIP), amoxicillin/clavulanic acid (AMC), cefotaxime/clavulanic acid (CTC), ceftazidime/clavulanic acid (CCV), polymyxin E (CT), polymyxin B (PB), minocycline (MIN), amikacin (AK), aztreonam (AZM), cefepime (FEP), meropenem (MEM), levofloxacin (LVX), doxycycline (DOX), kanamycin (KAN), streptomycin (STR), and gemifloxacin (GEM) using the reference broth microdilution method with custom plates. The Clinical & Laboratory Standards Institute (CLSI) breakpoints were used to assess the results. The *E. coli* ATCC 25922 strain was used for quality control.

### PCR Amplification and DNA Sequencing

All of the third-generation cephalosporin-resistant isolates were analyzed using PCR assays for the presence of extended-spectrum β-lactamase (ESBL) genes containing *bla*_CTX-M-1/9_ groups, *bla*_OXA_, *bla*_TEM_, *bla*_SHV_, and *bla*_CMY_. PCR amplification of QRDRs of *gyrA*, *gyrB*, *parC*, and *parE* and plasmid-borne quinolone resistance (PMQR) determinants (*qnrA*, *qnrB*, *qnrD*, *qnrS*, and *aac(6′)-Ib-cr*) were performed on the ciprofloxacin resistant isolates. Then PCR amplification was conducted to detect the plasmid-borne colistin (polymixin E)-resistant *mcr-1* gene in all isolates. The primers used for the abovementioned PCR assays are shown in Table 1. Amplification of the antibiotic resistance genes used the following temperature conditions: pre-denaturation at 95°C for 5min, followed by 30cycles of 95°C for 30s, annealing temperature for 30s and 72°C for 1min, with a final extension step at 72°C for 5min. The PCR products were analyzed by electrophoresis on 2.0% agarose. All of the PCR products were sequenced and then analyzed by DNAstar and the sequences were compared with the reference sequences from NCBI GenBank.

**Table 1:** Primers for the PCR detection of antimicrobial resistance determinants.

Target	Primer sequence (5'–3')	Annealing temperature (°C)	Amplicon size (bp)
QRDR of topoisomerase genes			
gyrAF	TCT CCG AGA TGG CCT GAA GC	55	347
gyrAR	TGC CGT CAT AGT TAT CCA CG		
gyrBF	CAA ACT GGC GGA CTG TCA GG	55	345
gyrBR	TTC CGG CAT CTG ACG ATA GA		
parCF	CTA TGC GAT GTC AGA GCT GC	55	275
parCR	TGA CCG AGT TCG CTT AAC AG		
parEF	GAC CGA GCT GTT CCT TGT GG	60	492
parER	GCG TAA CTG CAT CGG GTT CA		
PMQR			
qnrAF	GAG GAT TTC TCA CGC CAG GA	60	575
qnrAR	TGC CAG GCA CAG ATC TTG AC		
qnrB1F	GAT CGT GAA AGC CAG AAA GG	55	468
qnrB1R	ACG ATG CCT GGT AGT TGT CC		
qnrB2F	GTT GGC GAA AAA ATT GAC AGA A	57	451
qnrB2R	TTT GCA AGG CGT CAA ACT GG		
qnrCF	GGG TTG TAC ATT TAT TGA ATC	47	446
qnrCR	TCC ACT TTA CGA GGT TCT		
qnrDF	CGA GAT CAA TTT ACG GGG AAT A	60	581
qnrDR	AAC AAG CTG AAG CGC CTG		
qnrSF-1	GGA AAC CTA CAA TCA TAC ATA TCG GC	55	530
qnrSR-1	TAA ATT GGC ACC CTG TAG GC		
qnrSF-2	ATG GAA ACC TAC CGT CAC AC	55	638
qnrSR-2	ATA CCC AAC GCT TCG AGA AG		
aac(6*'*)-IB-crF	GCA ACG CAA AAA CAA AGT TAG G	47	560
aac(6*'*)-IB-crR	GTG TTT GAA CCA TGT ACA		
qepAF	GCA GGT CCA GCA GCG GGT AG	63	217
qepAR	CTT CCT GCC CGA GTA TCG TG		
β-lactamases			
bla_TEM_F	TCG GGG AAA TGT GCG	60	1,080
bla_TEM_R	TGC TTA ATC AGT GAG GCA CC		
bla_SHV_F	GCC TTT ATC GGC CTT CAC TCA AG	60	767
bla_SHV_R	TTA GCG TTG CCA GTG CTC GAT CA		
bla_CTX-M-1group_F	CAG CGC TTT TGC CGT CTA AG	60	867
bla_CTX-M-1group_R	GGC CCA TGG TTA AAA AAT CAC TGC		
bla_CTX-M-2group_F	CTC AGA GCA TTC GCC GCT CA	60	827
bla_CTX-M-2group_R	CCG CCG CAG CCA GAA TAT CC		
bla_CTX-M-9group_F	GTT ACA GCC TTC GGC GAT GAT TC	60	860
bla_CTX-M-9group_R	GCG CAT GGT GAC AAA GAG AGT GCA A		
bla_CMY-2_F	ATG ATG AAA AAA TCG TTA TGC T	50	1,126
bla_CMY-2_R	ATT GCA GCT TTT CAA GAA T		
Colistin-resistance genes			
mcr-1F	CGG TCA GTC CGT TTG TTC	60	308
mcr-1R	CTT GGT CGG TCT GTA GGG		

### Pulsed Field Gel Electrophoresis

The pulsed field gel electrophoresis (PFGE) results were taken from the routine surveillance data. PFGE was performed according to the PulseNet protocol for *Salmonella*, using *Xba*I as the restriction enzyme (TaKaRa Biotechnology, Dalian, China). The cluster analysis was performed with Bionumerics 5.0 (Applied Maths NV, Sint-Martens-Latem, Belgium) using the Dice similarity coefficient and UPMGA (unweighted pair group method using average linkages) dendrogram type (optimization 0.50%, position tolerance 1.50%).

### Multiloci Sequence Typing

The seven housekeeping gene sequences of *Salmonella* were amplified by PCR to determine the allelic differences and to analyze the sub-evolution relationship of the different strains. The primer sequences, amplification lengths and annealing temperature of the seven pairs of housekeeping sequences (aroC, dnaN, hemD, hisD, purE, sucA, and thrA) are shown in the previous reports. The PCR products were sequenced (Shanghai Biotechnology Co., Ltd.) and determination of the MLST was performed *in silico* using online tools.^1^

### Conjugation Assays

To test the host range and the transfer ability of each plasmid, conjugation assays were performed using the *E. coli* J53 strain as the recipient. Transfer of the colistin-resistance determinant by conjugation was assayed on LB agar plates with a donor:recipient ratio of 2:1, using *E. coli* J53 (sodium azide-resistant *E. coli*) as the recipient. After incubation at 37°C for 24h, transconjugants were selected on LB agar supplemented with colistin (2μg/ml) and sodium azide (180μg/ml). Initial species identification and subsequent antimicrobial susceptibility tests were conducted by using the VitekMS system. The transformants were regarded as transconjugants when they exhibited resistance to colistin and harbored the *mcr-1* gene.

### Plasmid Analysis

Isolates selected containing the *mcr-1* gene were subjected to further plasmid analysis. In compatibility groups, the plasmids extracted from the transconjugants were subjected to PCR-based replicon typing. S1-PFGE and Southern blotting were conducted to isolate and locate the resistance plasmids. Briefly, the gel plugs embedded with *mcr-1*-positive isolates were digested with S1 nuclease (TaKaRa Biotechnology, Dalian, China) and the linear plasmids were separated by the CHEF-Mapper XA PFGE system (Bio-Rad) as described above. The plasmid DNA was transferred to positive-charged nylon membranes (Millipore, United States), and a DIG-labelled *mcr-1*-specific probe was hybridized with the plasmids according to the instructions of the DIG High Prime DNA Labeling and Detection StarterKit (Roche, United States).

## Results

### Nontyphoidal *Salmonella* Isolates From Human Patients in Jiangsu, China, From 2016 to 2017

**Figure 1:**
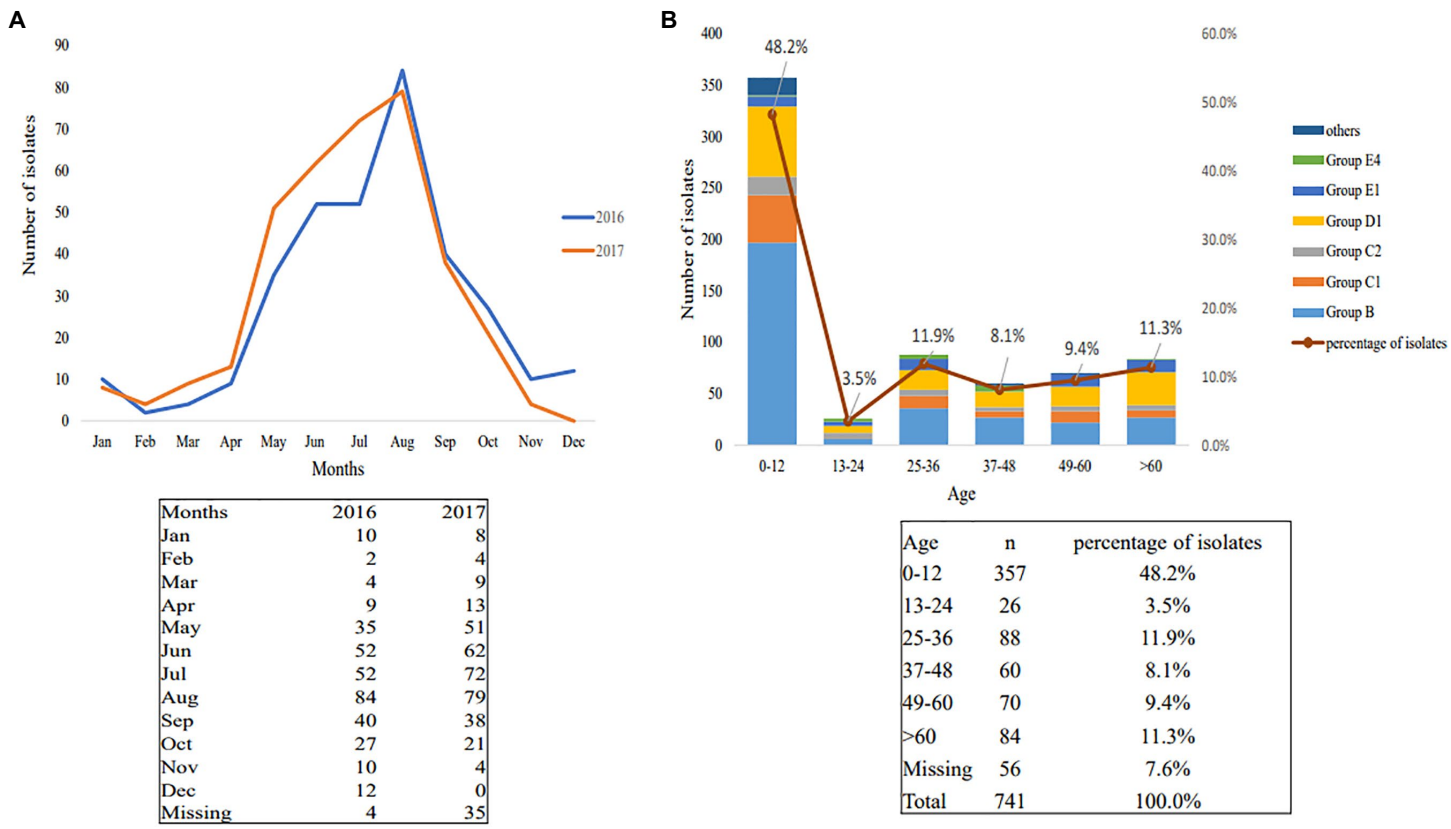
Season and age distributions of nontyphoidal *Salmonella* infection in 2016–2017. **(A)** Season distributions; **(B)** age distributions.

Between January 2016 and December 2017, 741 nontyphoidal *Salmonella* isolates were cultured from patients with diarrhea in Jiangsu, China. The age of the patients ranged from 4months to 79years (Figure 1B). Children under 12years of age were highly susceptible to nontyphoidal *Salmonella* infection, which accounted for 48.2% of all of the patients (*p*<0.05). The infections occurred mainly in summer and autumn (Figure 1A). In addition, the regional distribution shows that the three cities with the highest nontyphoidal *Salmonella* infection rates are Nanjing, Nantong, and Xuzhou (Table 2).

**Table 2:** Distribution of NTS infections between 2016 and 2017.

Regions	Number of isolates (%)		
	2016	2017	Total
Northern Jiangsu			
Xuzhou	45 (6.1)	42 (5.7)	87 (11.7)
Huai’an	37 (5.0)	27 (3.6)	64 (8.6)
Su qian	23 (3.1)	20 (2.7)	43 (5.8)
Lianyungang	15 (2.0)	17 (2.3)	32 (4.3)
Yancheng	4 (0.5)	0 (0)	4 (0.5)
Central Jiangsu			
Nanjing	51 (6.9)	99 (13.4)	150 (20.2)
Nantong	41 (5.53)	71 (9.6)	112 (15.1)
Taizhou	37 (5.0)	26 (3.5)	63 (8.5)
Yangzhou	4 (0.5)	15 (2.0)	19 (2.6)
Southern Jiangsu			
Zhengjiang	43 (5.8)	19 (2.6)	62 (8.4)
Wuxi	15 (2.0)	34 (4.6)	49 (6.6)
Suzhou	26 (3.5)	18 (2.4)	44 (5.9)
Changzhou	2 (0.3)	10 (1.4)	12 (1.6)
Total	343 (46.3)	398 (53.7)	741 (100)

**Figure 2:**
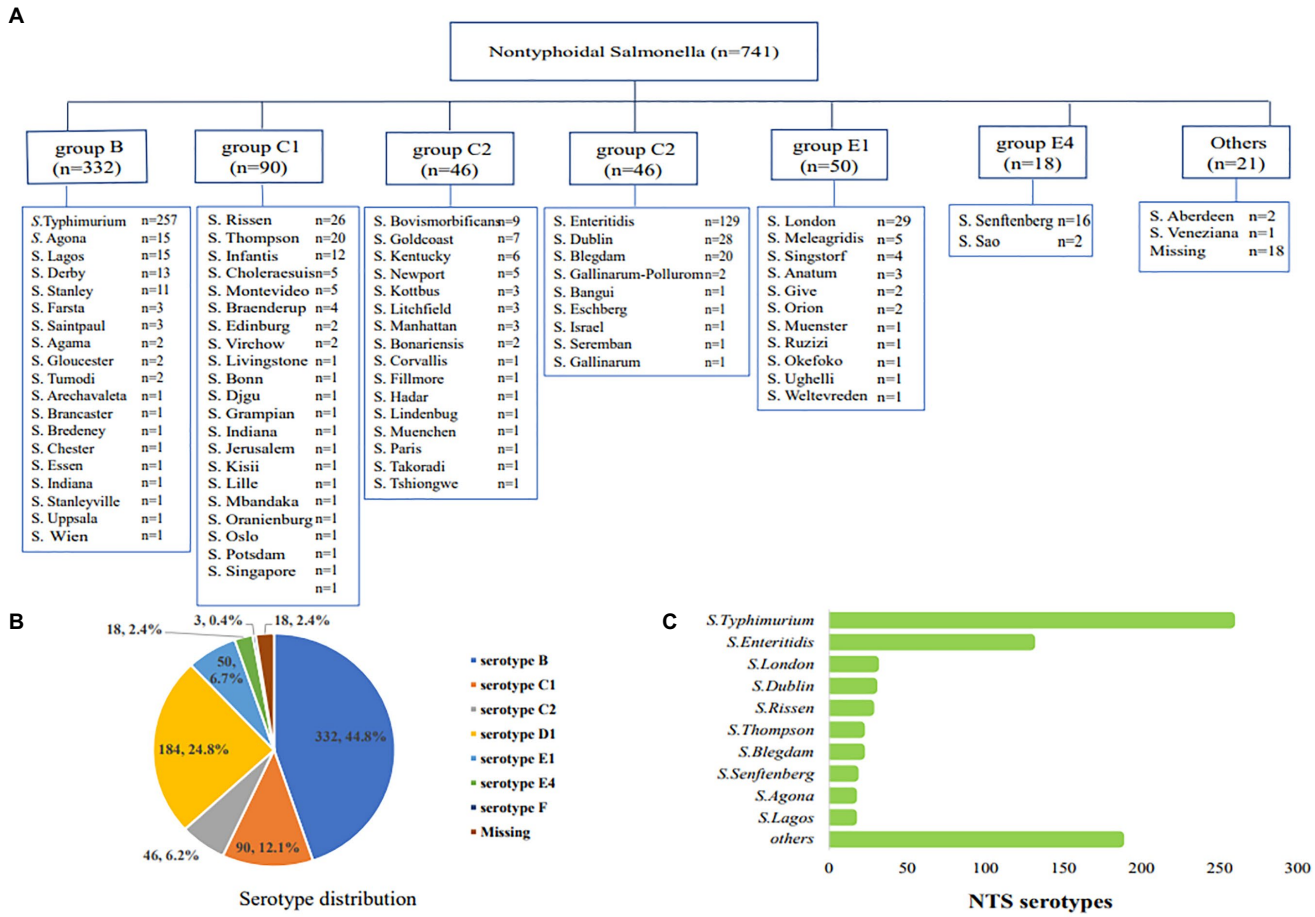
The serological distribution of nontyphoidal *Salmonella* from 2016 to 2017. **(A)** all of the serotype distribution of nontyphoidal *Salmonella* serotypes; **(B)** the distribution of serogroups; **(C)** the distribution of the top 10 nontyphoidal *Salmonella* serotypes.

Among the 741 cases of nontyphoidal *Salmonella*, 332 (44.8%) strains belonged to serogroup B, the most common serogroup, 184 (24.8%) strains to serogroup D, 136 (18.4%) strains to serogroup C, 68 (9.2%) strains to serogroup E, and 21 (2.8%) strains belonged to other serogroups (Figures 2A,B). The most common serotypes were *S.* Typhimurium (*n*=257, 34.7%), *S.* Enteritis (*n*=127, 17.4%), *S.* London (*n*=29, 3.9%), *S.* Dublin (*n*=28, 3.8%), and *S.* Rissen (*n*=26, 3.5%; Figure 2C).

### Antimicrobial Susceptibility Testing

Among the 741 isolates, only 15 (2.0%) were susceptible to all 30 antimicrobials. Resistance to ampicillin (72.3%) was the most common, followed by streptomycin (68.4%), sulfisoxazole (66.7%), and tetracycline (59.1%), the resistance rate of which exceeded 50% (Table 3). In addition, resistance to cefotaxime, ceftazidime and cefoxitin were found in 21.7, 10.5, and 9.6% of isolates, respectively. Notably, 45.2% (335/741) and 20.6% (153/741) of the isolates displayed resistance to nalidixic acid and ciprofloxacin, while 15 of the isolates showed co-resistance to quinolones and third-generation cephalosporins. More importantly, 5% (37/741) of the isolates displayed resistance to colistin. Among the MDR isolates, 200 (27.0%) showed the ACSSuT resistance pattern (defined as resistance to ampicillin, chloramphenicol, streptomycin, sulfamethoxazole, and tetracycline).

**Table 3:** Antimicrobial resistance of NTS isolates in Jiangsu from 2016 to 2017.

Antimicrobial agent	Nontyphoidal Salmonella serogroup/Number of resistant isolates (%)														All serotypes (*n*=741)	
	Group B (*n*=332)		Group C1 (*n*=90)		Group C2 (*n*=46)		Group D1 (*n*=184)		Group E1 (*n*=50)		Group E4 (*n*=18)		Others (*n*=21)			
	*n*	%	*n*	%	*n*	%	*n*	%	*n*	%	*n*	%	*n*	%	*n*	%
Pan-susceptible															15	2.0
Ampicillin	256	77.1	59	65.6	29	63.0	149	81.0	31	62.0	4	22.2	7	33.3	535	72.2
Ampicillin-salbactam	140	42.2	45	50.0	24	52.2	102	55.4	21	42.0	2	11.1	7	33.3	341	46.0
Tetracycline	251	75.6	60	66.7	28	60.9	50	27.2	35	70.0	3	16.7	11	52.4	438	59.1
Chloramphenicol	149	44.9	41	45.6	16	34.8	23	12.5	28	56.0	2	11.1	4	19.0	263	35.5
Trimethoprim/sulfamethoxazole	143	43.1	51	56.7	17	37.0	25	13.6	30	60.0	4	22.2	4	19.0	274	37.0
Aztreonam	40	12.0	11	12.2	3	6.5	32	17.4	2	4.0	1	5.6	2	9.5	91	12.3
Cefotaxime	72	21.7	27	30.0	8	17.4	43	23.4	7	14.0	0	0.0	4	19.0	161	21.7
Ceftazidime	28	8.4	18	20.0	2	4.3	25	13.6	4	8.0	0	0.0	1	4.8	78	10.5
Cefoxitin	29	8.7	22	24.4	6	13.0	8	4.3	4	8.0	0	0.0	2	9.5	71	9.6
Gentamicin	49	14.8	10	11.1	7	15.2	21	11.4	22	44.0	0	0.0	2	9.5	111	15.0
Nalidixic acid	114	34.3	21	23.3	17	37.0	158	85.9	13	26.0	4	22.2	8	38.1	335	45.2
Azithromycin	20	6.0	7	7.8	2	4.3	6	3.3	13	26.0	0	0.0	1	4.8	49	6.6
Ciprofloxacin	78	23.5	29	32.2	11	23.9	13	7.1	18	36.0	0	0.0	4	19.0	153	20.6
Amoxicillin-clavulanic acid	56	16.9	27	30.0	9	19.6	14	7.6	7	14.0	1	5.6	3	14.3	117	15.8
Sulfasalazine	251	75.6	61	67.8	27	58.7	107	58.2	35	70.0	5	27.8	8	38.1	494	66.7
Colistin	16	4.8	4	4.4	3	6.5	11	6.0	3	6.0	0	0.0	0	0.0	37	5.0
Polymyxin B	6	1.8	1	1.1	1	2.2	4	2.2	1	2.0	0	0.0	0	0.0	13	1.8
Kanamycin	61	18.4	11	12.2	9	19.6	33	17.9	8	16.0	2	11.1	3	14.3	127	17.1
Streptomycin	249	75.0	56	62.2	34	73.9	112	60.9	35	70.0	9	50.0	12	57.1	507	68.4
ACSSuT (MDR)															200	27.0

### PCR Detection of Antimicrobial Drug Resistance Genes and DNA Sequencing

**Figure 3:**
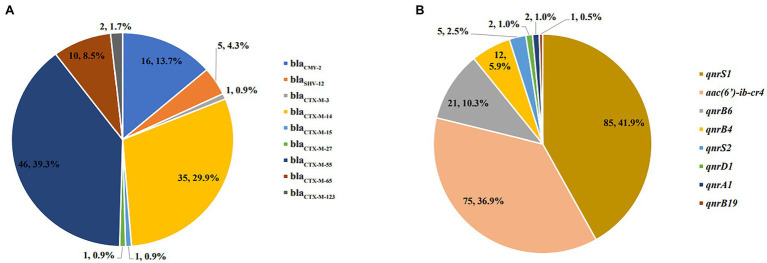
Distribution of plasmid-borne quinolone resistances (PMQRs) and extended-spectrum beta-lactamases (ESBLs) resistance genes of nontyphoidal *Salmonella* from 2016 to 2017. **(A)** ESBLs resistance genes of nontyphoidal Salmonella; **(B)** PMQRs resistance genes of nontyphoidal *Salmonella*.

Resistance genes including *bla*_OXA_, *bla*_SHV_, *bla*_CMY_, *bla*_CTX-M-1 group_, and *bla*_CTX-M-9 group_ in the 111 cephalosporin-resistance isolates were detected by PCR except *bla*_TEM_, for which the detection rate exceeded 72.5%. PCR screening revealed that 93 (83.8%), 16 (14.4%), and 5 (4.5%) isolates contained the *bla*_CTX-M-1/9_, *bla*_CMY_, and *bla*_SHV_ genes, respectively. All of the strains were negative for *bla*_OXA_. Sequencing of the PCR products revealed that *bla*_CTX-M-1 group_ included four subtypes: *bla*_CTX-M-55_ (*n*=46), *bla*_CTX-M-123_ (*n*=2), *bla*_CTX-M-15_ (*n*=1), and *bla*_CTX-M-3_ (*n*=1); the *bla*_CTX-M-9 group_ included three subtypes: *bla*_CTX-M-14_ (*n*=35), *bla*_CTX-M-65_ (*n*=10), and *bla*_CTX-M-27_ (*n*=1), among which *bla*_CTX-M-14_ and *bla*_CTX-M-55_ were the most common subtypes. *Bla*_SHV-12_ subtype and *bla*_CMY-2_ were detected in all five *bla*_SHV_ and 16 *bla*_CMY_ nontyphoidal *Salmonella* strains, respectively. Notably, two isolates contained *bla*_TEM-1_, *bla*_CTX-M-55_, and *bla*_CTX-M-65_ subtypes, one isolate contained *bla*_TEM-1_, *bla*_CTX-M-14_, and *bla*_CMY-2_ subtypes, one isolate contained *bla*_TEM-1_, *bla*_CTX-M-65_, and *bla*_SHV-12_, and one isolate concomitantly contained *bla*_TEM-1_, *bla*_CTX-M-3_, *bla*_CTX-M-65_, and *bla*_SHV-12_ (Figure 3A).

A total of 180 PMQR-positive strains were detected by PCR in 741 nontyphoidal *Salmonella* strains. Sequencing of the PCR products revealed that 44.3% of the *qnrS* genes included *qnrS1* (*n*=85) and *qnrS2* (*n*=5) subtypes and 16.7% of the *qnrB* genes included *qnrB6* (*n*=21), *qnrB4* (*n*=12), and *qnrB19* (*n*=1) subtypes. *qnrA1* (*n*=2) subtypes and *qnrD1* (*n*=2) and *aac (6′)-Ib-cr4* (*n*=75) subtypes were detected in all *qnrA*, *qnrD*, and *aac(6′)-Ib-cr* nontyphoidal *Salmonella* strains. We found that *qnrS1* and *aac(6′)-Ib-cr4* were the most common subtypes of PMQR-positive genes in Jiangsu, China (Figure 3B).

In addition, the PCR results showed that three out of the 37 colistin-resistant isolates [minimum inhibitory concentration (MIC)≥4μg/ml] harbored plasmid-borne *mcr-1* genes, while the other five *mcr-1* genes were found in colistin-intermediate resistance nontyphoidal *Salmonella* isolates (MIC=2μg/ml).

We identified 449 isolates with intermediation and resistance to ciprofloxacin, and 185 of them contained at least one *gyrA* mutation, which mainly occurred at codons 83/87 (S83F/Y; D87Y/N/G). In addition, 45 of them contained at least one *parC* mutation, which mainly occurred at codons 57/80 (T57S; S80I/R). No point mutations in *gyrB* were found among the sequences of the QRDRs. Among the 47 isolates with one or more mutation in the *gyrA*, *parC*, and *parE* genes, one or two PMQR genes were detected, and for two isolates, we simultaneously detected *gyrA*, *parC*, and *parE* mutations and plasmid-borne PMQR resistance genes (Supplementary Table S1).

Among the 80 nontyphoidal *Salmonella* isolates with a phenotype showing concomitant resistance to ciprofloxacin and cefotaxime, 40 isolates contained ESBL and PMQR genes and 17 isolates harbored three types of antimicrobial resistant genes, like *aac(6′)-Ib-cr4*/*bla*_CTX-M_/*bla*_TEM-1_ (*n*=4) and *qnrS1*/*bla*_TEM-1_/*bla*_CMY-2_ (*n*=4). Two isolates contained four types of antimicrobial-resistance genes: *aac(6′)-ib-cr4*/*bla*_TEM-1_/*bla*_CTX-M-14_/*mcr-1* and *qnrS1*/*aac(6′)-ib-cr4*/*bla*_TEM-1_/*bla*_CTX-M-65_. One *Salmonella typhimurium* isolate contained six types of antimicrobial-resistance genes: *qnrS1*/*qnrS2*/*aac(6′)-ib-cr4*/*bla*_TEM-1_/*bla*_CTX-M-14_/*mcr-1*. In addition, 11 isolates that contained QRDR mutations also harbored the ESBL and PMQR genes at the same time (Supplementary Table S2; Table 4).

**Table 4:** Summary of phenotypes of NTS isolates showing concurrently resistance to ciprofloxacin and cefotaxime and their corresponding resistance genes in 2017.

Sample ID	Serotype	Year	City	MIC (mg/L)		QRDR amino acid substitutionsa			Plasmid-mediated resistance	
				CTX	CIP	gyrA	parC	parE	PMQR	*β-lactams*
SA17003	S.Thompson	2017	Changzhou	>8	8	/	/	/	/	*TEM-1, CMY-2*
SA17005	S.Thompson	2017	Changzhou	>8	8	/	/	/	/	*TEM-1, CMY-2*
SA17012	S.Enteritidis	2017	Huai’an	>8	1	/	/	/	/	*TEM-1*
SA17036	S.Blegdam	2017	Huai’an	>8	32	/	/	/	/	*TEM-1*
SA17040	S.Typhimurium	2017	Huai’an	>8	1	D87N	/	/	/	*TEM-1*
SA17080	S.Typhimurium	2017	Nanjing	>8	8	S83F/D87G	T57S	/	/	*TEM-1*
SA17082	S.Thompson	2017	Nanjing	>8	8	/	/	/	qnrS1	*TEM-1*
SA17084	S.Enteritidis	2017	Nanjing	>8	1	D87Y	/	/	/	*TEM-1, CTX-M-14*
SA17102	S.Thompson	2017	Nanjing	>8	8	/	/	/	*qnrS1, qnrS2*	*TEM-1*
SA17108	S.Oritamerin	2017	Nanjing	>8	8	/	/	/	*qnrS1*	*TEM-1*
SA17127	S.Enteritidis	2017	Nantong	8	1	D87G	/	/	/	*TEM-1*
SA17128	S.Rissen	2017	Nantong	4	1	/	T57S	/	/	*TEM-1, CTX-M-55*
SA17147	S.Derby	2017	Nantong	>8	2	S83A	T57S	L440R/R508K/N516H/A545E	*qnrS2*	*TEM-1*
SA17148	S.Rissen	2017	Nantong	>8	1	/	/	/	/	*TEM-1*
SA17152	S.Thompson	2017	Nantong	>8	4	/	/	/	*qnrS1*	*TEM-1*
SA17155	S.Thompson	2017	Nantong	>8	4	/	/	/	*qnrS1*	*TEM-1*
SA17175	S.London	2017	Suqian	>8	1	/	/	/	*qnrS1, qnrB6, aac(6')-ib-cr4*	*TEM-1*
SA17179	S.Typhimurium	2017	Suqian	>8	>32	/	/	/	/	*TEM-1*
SA17185	S.Typhimurium	2017	Suqian	>8	>32	/	/	/	/	*TEM-1*
SA17191	S.Typhimurium	2017	Suqian	>8	1	/	/	/	*qnrB4, aac(6')-ib-cr4*	*TEM-1*
SA17211	S.Typhimurium	2017	Taizhou	>8	4	/	/	/	*qnrS1, qnrS2, aac(6')-ib-cr4*	*TEM-1, CTX-M-14, mcr-1*
SA17217	S.Infantis	2017	Taizhou	>8	4	/	/	/	*qnrS1*	*TEM-1*
SA17246	S.Thompson	2017	Wuxi	>8	4	/	/	/	*aac(6')-ib-cr4*	*TEM-1, CMY-2*
SA17251	S.Thompson	2017	Xuzhou	>8	8	/	/	/	*qnrS1*	*TEM-1, SHV-12*
SA17257	S.Typhimurium	2017	Xuzhou	>8	8	S83A	/	/	/	*TEM-1*
SA17263	S.Typhimurium	2017	Xuzhou	>8	1	/	/	/	/	*TEM-1, CTX-M-65*
SA17276	S.Indiana	2017	Xuzhou	>8	32	/	/	/	/	*TEM-1*
SA17279	S.Typhimurium	2017	Xuzhou	>8	1	D87Y	/	/	/	*TEM-1, CTX-M-55*
SA17283	S.Typhimurium	2017	Xuzhou	>8	4	/	/	/	*qnrB4*	*TEM-1, SHV-12*
SA17290	S.Enteritidis	2017	Xuzhou	>8	1	/	/	/	/	*TEM-1*
SA17295	S.Choleraesuis	2017	Yangzhou	>8	32	S83I/D87G/K154R	/	A512T/N516E/A545T	*qnrS1, aac(6')-ib-cr4*	*TEM-1, CTX-M-65*
SA17324	S.Thompson	2017	Zhenjiang	>8	4	/	/	/	/	*TEM-1, CMY-2*
SA17332	S.Enteritidis	2017	Nanjing	>8	32	/	/	/	/	*TEM-1*
SA17337	WT	2017	Nanjing	>8	8	/	/	/	/	*TEM-1*
SA17340	S.Derby	2017	Nantong	8	2	/	T57S	/	*qnrS2*	*TEM-1*
SA17348	S.Thompson	2017	Nantong	>8	8	/	/	/	*qnrS1*	*TEM-1, CMY-2*
SA17349	S.London	2017	Nantong	>8	2	/	T57S	/	*aac(6')-ib-cr4*	*TEM-1*
SA17360	S.London	2017	Nantong	>8	1	/	T57S	/	*aac(6')-ib-cr4*	*TEM-1*
SA17390	WT	2017	Nanjing	>8	32	S83L/D87G	/	R508K/N516H/A545E	*qnrS1*	*TEM-1, CTX-M-55*
SA17395	S.Kentucky	2017	Nanjing	>8	8	S83F/D87G	T57S/S80I	/	/	*TEM-1, CTX-M-14*
SA17407	S.Lindenbug	2017	Xuzhou	>8	8	/	/	/	/	*TEM-1*
SA17408	S.Typhimurium	2017	Xuzhou	>8	1	/	/	/	/	*TEM-1*

### Epidemiology of *mcr-1*-Harboring Nontyphoidal *Salmonella* Isolates

**Figure 4:**
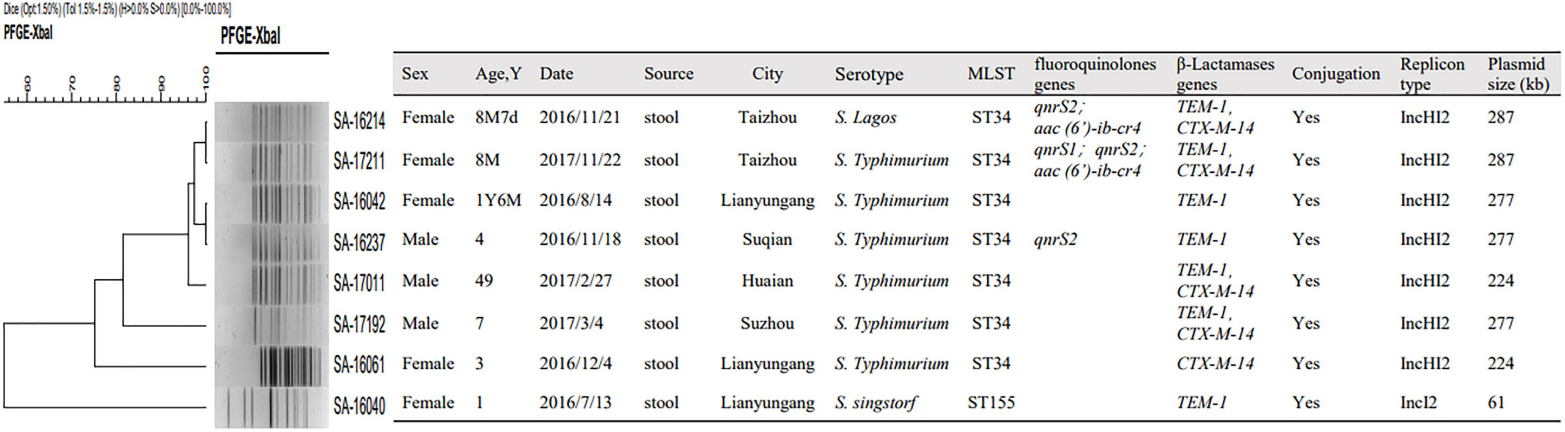
Pulsed field gel electrophoresis (PFGE), multiloci sequence typing (MLST), resistance gene, conjugation experiment, replicon typing and plasmid distribution of nontyphoidal *Salmonella* carrying *mcr-1* from 2016 to 2017.

Over the course of the study, eight of nontyphoidal *Salmonella* were found to harbor the *mcr-1* gene. Among the eight patients infected with *mcr-1*-positive nontyphoidal *Salmonella*, seven were aged under 7years old and the other was 49. Among these isolates, five strains were detected in Northern Jiangsu including Lianyungang (*n*=3), Suqian (*n*=1), and Huai’an (*n*=1), two were from Taizhou (*n*=2) and one was from Suzhou (*n*=1). In addition, two different serotypes were identified among the eight isolates mentioned above, including six *S.* Typhimurium, one *S.* Lagos, and one *S.* Sinstorf (Figure 4).

### Molecular Typing of *mcr-1* Harboring Nontyphoidal *Salmonella* Isolates

Among eight *mcr-1*-positive nontyphoidal *Salmonella* isolates, two STs were identified. ST 34 was the most prevalent, accounting for 87.5% (*n*=7), followed by ST 155 (*n*=1). *Salmonella* ST155 was found in *mcr-1* positive patients for the first time. PFGE typing revealed that five isolates with the same ST type showed the same band pattern except for ST155 *Salmonella* collected in Lianyungang and ST34 *S.* Typhimurium in Suzhou and Lianyungang (Figure 4).

### Characteristics of Plasmids Harboring the *mcr-1* Gene

**Figure 5:**
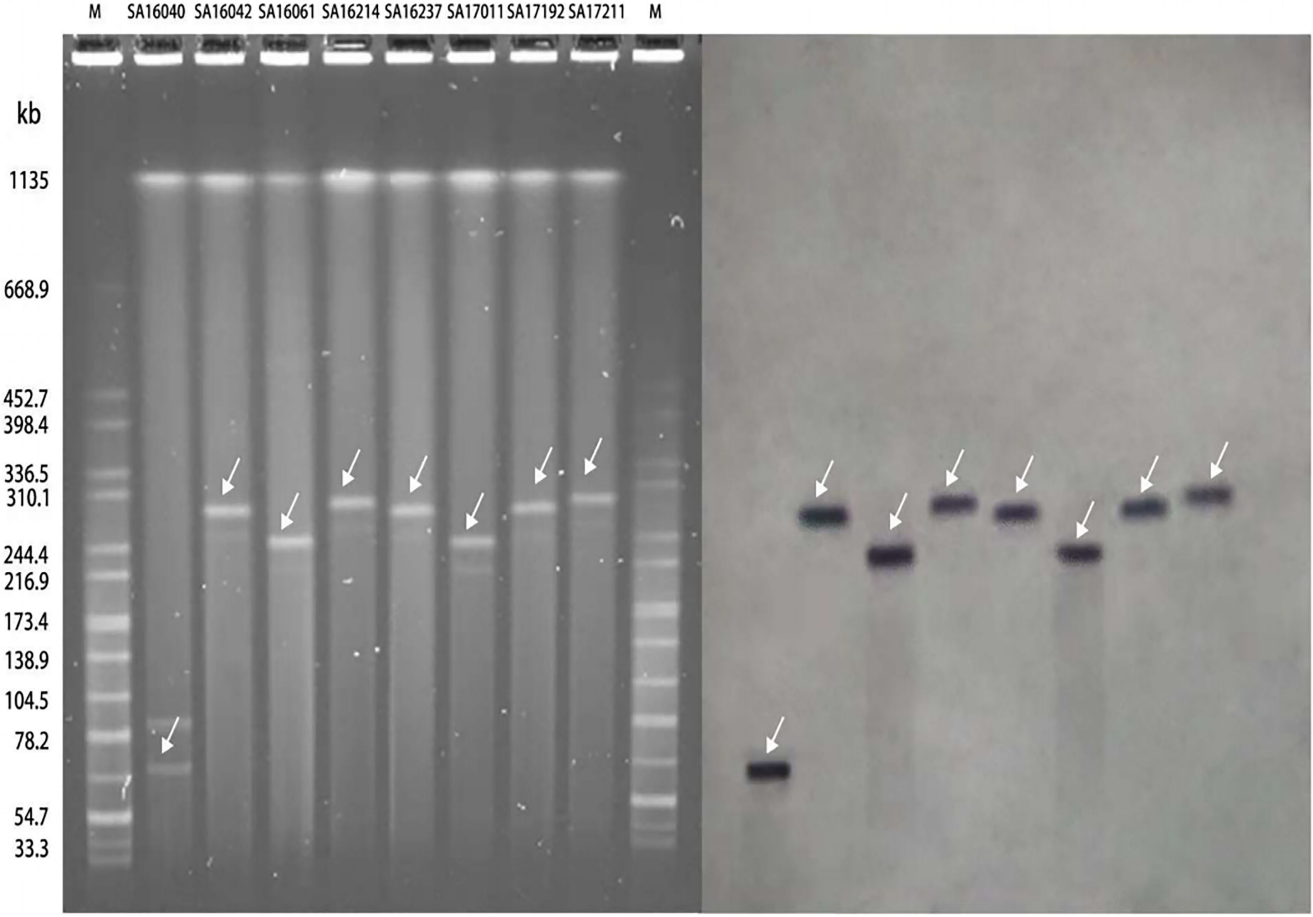
Analysis of the results of S1-PFGE and DNA hybridization of *mcr-1* positive nontyphoidal *Salmonella* from 2016 to 2017.

All of the plasmids harboring the *mcr-1* gene from eight selected nontyphoidal *Salmonella* isolates were successfully transferred to *E. coli* J53, and the transconjugants exhibited resistance to colistin and cephalosporins when the parent *mcr-1*-harbouring nontyphoidal *Salmonella* exhibited resistance. This shows that the *mcr-1* gene could be expressed and function in the transconjugants. In addition, the transconjugants showed high resistance to cefotaxime, suggesting that genes co-transfer occurred during the conjugation process (Table 5). S1-PFGE and Southern blotting revealed that the among the eight *mcr-1*-haboring plasmids, the replicon types IncI2 and IncHI2 were detected in one and seven plasmids, respectively (Figure 5). The earliest isolation of a strain carrying the IncI2 type *mcr-1* plasmid *S.* Sinstorf occurred in Lianyungang, Jiangsu.

**Table 5:** Antimicrobial susceptible patterns and characteristics of eight selected mcr-1-producing NTS (μg/ml).

Patient ID	AMP	SAM	TET	CHL	SXT	CFZ	CTX	CAZ	CFX	GEN	NAL	AZI	SUL	CIP	AMC	CT	AZT	FEP	KAN	STR
SA16040	>64	32/16	32	>64	>8/152	4	≤0.25	≤0.5	≤2	>32	8	32	>512	1	32/16	4	≤1	0.5	≤8	>32
SA16042	>64	64/32	>32	>64	>8/152	>16	>8	2	8	8	>64	4	>512	16	32/16	4	16	16	>64	>32
SA16061	>64	64/32	32	>64	>8/152	>16	>8	≤0.5	4	≤1	8	≤2	>512	≤0.03	8/4	4	≤1	1	>64	8
SA16214	>64	64/32	>32	>64	>8/152	>16	>8	1	≤2	8	32	4	>512	4	32/16	4	16	16	16	>32
SA16237	>64	64/32	>32	>64	>8/152	>16	>8	4	≤2	16	>64	4	>512	8	32/16	2	16	16	>64	>32
SA17011	>64	32/16	>32	>64	1/19	>16	>8	4	≤2	16	32	4	>512	0.125	16/8	2	8	16	≤8	>32
SA17192	>64	>64/32	2	>64	>8/152	>16	>8	8	16	>32	64	16	>512	0.25	16/8	2	>32	>16	>64	>32
SA17211	>64	64/32	>32	>64	>8/152	>16	>8	2	≤2	16	64	≤2	>512	4	32/16	2	8	16	16	>32
EJ53	8	4/2	2	4	≤0.25/4.75	2	≤0.25	≤0.5	≤2	≤1	≤4	≤2	≤32	≤0.03	4/2	≤0.5	≤1	≤0.25	≤8	≤4
SA16040-EJ53	8	8/4	2	4	≤0.25/4.75	1	≤0.25	≤0.5	≤2	≤1	≤4	≤2	≤32	≤0.03	4/2	4	≤1	≤0.25	≤8	≤4
SA16042-EJ53	>64	32/16	2	>64	>8/152	>16	8	≤0.5	≤2	≤1	32	≤2	>512	2	16/8	4	2	2	>64	8
SA16061-EJ53	>64	8/4	2	>64	>8/152	>16	4	≤0.5	4	≤1	8	≤2	>512	≤0.03	8/4	4	≤1	1	>64	8
SA16214-EJ53	>64	32/16	32	>64	>8/152	>16	8	≤0.5	≤2	≤1	32	≤2	>512	2	16/8	4	≤1	2	≤8	≤4
SA16237-EJ53	>64	32/16	2	>64	>8/152	>16	>8	1	4	2	16	≤2	>512	1	16/8	4	2	2	>64	32
SA17011-EJ53	>64	8/4	2	>64	0.5/9.5	>16	8	≤0.5	≤2	≤1	16	≤2	>512	0.06	8/4	4	≤1	1	≤8	8
SA17192-EJ53	>64	8/4	2	64	>8/152	>16	4	≤0.5	≤2	≤1	8	8	>512	≤0.03	8/4	2	≤1	1	>64	≤4
SA17211-EJ53	>64	32/16	32	>64	>8/152	>16	8	≤0.5	≤2	≤1	32	≤2	>512	2	16/8	4	≤1	2	≤8	≤4

The antibiotic resistance genes in these *mcr-1*-harbouring nontyphoidal *Salmonella* were analyzed. All of these nontyphoidal *Salmonella* carried resistance genes other than *mcr-1*. Five of the seven nontyphoidal *Salmonella* with IncHI2 type *mcr-1* plasmids simultaneously carried the *bla*_CTX-M-14_ gene, whereas none of the IncI2 plasmids were found to harbor other antibiotic resistance genes except *bla*_TEM-1_. Additionally, one isolate with the IncHI2 *mcr-1* plasmid was confirmed to harbor *mcr-1* and *qnrS2*, while two isolates with the IncHI2 type *mcr-1* plasmid harbored both PMQRs and ESBLs (one with *qnrS2*/*aac(6′)-ib-cr4* and *bla*_TEM-1_/*bla*_CTX-M-14_; one with *qnrS1*/*qnrS2*/*aac(6′)-ib-cr4* and *bla*_TEM-1_/*bla*_CTX-M-14_; Figure 4).

## Discussion

Human and animal salmonellosis is a symptomatic infection caused by bacteria of the *Salmonella* enterica, mainly occurring in many low- and middle-income countries (Reddy et al., 2010). It is estimated that it causes approximately 153 million gastroenteritis cases and 57,000 deaths worldwide every year (Majowicz et al., 2010), and more than half of these diseases and deaths occur in Africa (Ao et al., 2015). Our results showed that the detection rate of nontyphoidal *Salmonella* in Jiangsu from 2016 to 2017 is relatively high, which should arouse clinical concern. Our results found that the number of nontyphoidal *Salmonella* isolated from Central Jiangsu and Northern Jiangsu is higher than that from Southern Jiangsu. The highest isolation rate was in Nanjing, which may be related to the large local population and its frequent movements.

Six serovar including serotype B, C1, C2, D1, E1, and E4 were found in 741 nontyphoidal *Salmonella* strains. *Salmonella* Typhimurium and *S.* Enteritidis were the most common subtypes, consistent with the most widely reported serotypes of *S.* Typhimurium and *S.* Enteritidis in Africa (Mabey et al., 1987; Gordon et al., 2008). Additionally, our study also identified 58 other serovars. The continuous emergence of rare serovars in recent years should arouse great concern in Jiangsu, China.

The introduction of fluoroquinolones and third-generation cephalosporins in the 1980s greatly reduced the mortality from salmonellosis. However, fluoroquinolone-resistant *Salmonella* appeared in the 1990s. In areas where the infection is not endemic, such as in the United States and Canada, the resistance rate of nalidixic acid reached 40 and 80% in 2004 and 2006, respectively (Demczuk et al., 2010), while the resistance rate of ciprofloxacin in the United Kingdom in 2006 was 70% (Threlfall et al., 2008). We conducted drug-resistant phenotype monitoring of nontyphoidal *Salmonella* in Jiangsu from 2016 to 2017 and found that the drug-resistant rate of nalidixic acid was 45.2%, consistent with the resistance level in the United States. The drug resistance rate to ciprofloxacin was significantly higher among the *S.* Typhi in Jiangsu in the past 5years (Qian et al., 2020).

The appearance of fluoroquinolone resistance has aroused our interest in the study of its resistance mechanism. Fluoroquinolone resistance in *Salmonella* is usually mediated through mutations in chromosomal sites or through plasmid-borne PMQR resistance genes. Early studies of *Salmonella* found that increases of the MIC was related to the accumulation of known chromosomal mutations (Murray et al., 2012; Song et al., 2013). Our study found that the combined mutation detection rate of *gyrA* gene 83, 87 and *parC* gene 57 in ciprofloxacin resistant strains was the highest, which confirmed that the multiple-site QRDR combined mutations are a possible cause of ciprofloxacin resistance. Additionally, our sequencing results also found that nontyphoidal *Salmonella* harboring *qnr* and *aac(6′)-Ib-cr* mainly appeared in strains with reduced ciprofloxacin susceptibility, which suggested that PMQRs genes may mediate low-level ciprofloxacin-resistance.

The emergence of *Salmonella* that is resistant to third-generation cephalosporins such as ceftriaxone and cefotaxime indicates another important public health problem. Ever since the third-generation cephalosporin drugs have been used in the clinic, ESBLs or AmpC-type β-lactamases mediated resistance has been detected in nontyphoidal *Salmonella*. Cephalosporin resistance has been reported in Southeast Asia (Koh et al., 2008), while in Thailand, *Salmonella* with *bla*_CMY_ and *bla*_CTX-M_ enzymes has also been described (Sirichote et al., 2010; Pornruangwong et al., 2011). Our research found that the resistance rate of nontyphoidal *Salmonella* to cefotaxime was more than 20%, significantly higher than that of *S.* Typhi (Qian et al., 2020). Coinciding with domestic and international reports, *bla*_CTX-M_-type and *bla*_CMY-2_ genes were simultaneously detected in nontyphoidal *Salmonella*, the majority of which existed in cefotaxime-resistant strains. If ESBL-producing nontyphoidal *Salmonella* becomes common, treatment options may become very scarce. Therefore, timely detection is essential to control the spread of β-lactamase among nontyphoidal *Salmonella*.

With the continuous emergence of third-generation cephalosporin and fluoroquinolone resistant *Salmonella* strains, plasmid-borne resistance gene transmission has been found to play an extremely important role. In recent years, 40 strains of nontyphoidal *Salmonella* co-existing with ESBL and PMQR genes have been discovered. The complexity and diversity of nontyphoidal *Salmonella* resistance genes make clinical treatment tricky.

Colistin is a class of cationic cyclic peptide antibiotics synthesized with a non-ribosomal hydrophobic tail (Dixon and Chopra, 1986). Its mechanism of killing Gram-negative pathogens relies on its ability to destroy membranes through polar hydrophobic interactions. The potential nephrotoxicity and neurotoxicity of colistin prevent its widespread use in the clinic (Li et al., 2006; Baron et al., 2016). At present in Europe, colistin has been widely used in animal production to treat many animal infections caused by *Enterobacteriaceae* (Catry et al., 2015). Moreover, in Asian countries, colistin is widely used as a growth promoter to improve animal production (Kempf et al., 2016). With its long-term use, many bacterial species have developed resistance to colistin. Since Liu et al., first reported the discovery of the mobile colistin resistance gene *mcr-1* in Chinese pig and human *E. coli* isolates in 2015, the *mcr-1* gene has been reported from samples collected in five continents and more than 40 countries, which means that it plays an important role in colistin resistance. As the last line of defense against multi-drug resistant bacteria, colistin has attracted worldwide attention. In our study, 5% (37/741) of clinical nontyphoidal *Salmonella* isolates from Jiangsu between 2016 and 2017 showed colistin resistance (MIC≥4μg/ml).

In China, colistin first started to be used in food-producing animals as early as the 1980s. Shen et al., found that the spread of *mcr-1* in *E. coli* isolates of chicken origin in China increased from 5.2% in 2009 to 30.0% in 2014. This coincides with the fact that China introduced colistin into agriculture at the beginning of 2000, and by the end of 2015, China had become one of the world’s largest users of agricultural colistin (Shen et al., 2016). Among *Salmonella*, *S.* Typhimurium is one of the most common serotypes harboring the *mcr-1* gene. The *mcr-1* gene was described for the first time by analyzing the complete genome sequence of *Salmonella* available in GenBank, including the identification of strains carrying the *mcr-1* plasmid among 10 clinical *Salmonella* isolates submitted between 2012 and 2015 (Doumith et al., 2016). Subsequently, the *mcr-1* gene has been reported in *Salmonella* strains isolated from food, animal and clinical specimens from Europe, the United States and China (Anjum et al., 2016; Campos et al., 2016; Yang et al., 2016). We retrospectively analyzed the prevalence and molecular characteristics of the *mcr-1* gene in nontyphoidal *Salmonella* isolates and found that eight *mcr-1* positive nontyphoidal *Salmonella*.

The serotype is one of the important phenotypic characteristics of *Salmonella* and serovar analysis is an important traceability analysis method for *Salmonella* (Soler-Garcia et al., 2014). From the perspective of *Salmonella* serotype analysis, the *mcr-1* positive strains are mainly *S.* Typhimurium (6/8). Notedly, a case of a patient with *S.* Sinstorf carrying *mcr-1* has not been reported previously, indicating the diversity of *mcr-1* transmission. The majority of patients infected with the *mcr-1* strain are children, accounting for 87.5% (7/8), which is consistent with the data reported in Shanghai by Lu et al. (2019). The typing of *Salmonella* is helpful for the traceability analysis of pathogenic microorganisms, and it is of great significance to the prevention and control of *Salmonella* and risk assessment.

Multiloci sequence typing is a bacterial typing method based on the determination of the nucleotide sequence with high resolution and it is suitable for molecular epidemiology research and molecular evolution analyses. Each ST represents a separate set of nucleotide sequence information. Closely related strains have the same ST or only a few different gene sites of the ST type. In China, the most common ST of *Salmonella*, especially MDR *Salmonella*, is ST34, and this is also common in Europe. Previous reports showed that most of the ST34 *Salmonella* strains carrying the *mcr-1* gene were isolated from animals, while we found that six strains of *S.* Typhimurium and one strain of *S.* Lagos belonged to the ST34, indicating the widespread spread of ST34 nontyphoidal *Salmonella* harboring the *mcr-1* gene in animals and humans. The widespread existence of this clone poses a great threat to the prevention and control of clinical *S.* Typhimurium infection. In our study, a strain of *Salmonella* ST155 carrying *mcr-1* was detected from a patient. ST155, as a new ST type carrying the *mcr-1* gene, was reported in humans for the first time. In addition, the PFGE results of our study showed that five *mcr-1* positive nontyphoidal *Salmonella* strains from Taizhou, Huai’an, Suqian, and Lianyungang had the same PFGE type, suggesting that the clonal spread of the *mcr-1* gene has occurred in the Central and Northern of Jiangsu, which means that infection control should be carried out at the same time in multiple hospitals and multiple regions in Jiangsu.

Since the initial detection of *mcr-1* in the IncI2 plasmid pHNSHP45 (Liu et al., 2016), the diversity of *mcr-1* harboring plasmid libraries has continued to expand. Analysis of the data related to the *mcr-1* gene in GenBank showed that IncI2, IncX4, and IncHI2 are the most common plasmids carrying the *mcr-1* gene. Other replicon types such as IncP, IncHI1, IncFII, IncFI, IncFIB, F:A−:B+, IncY, IncK and phage-like plasmids have also been reported (Nordmann et al., 2016; Xavier et al., 2016; Zurfluh et al., 2016; Li et al., 2017; Zhang et al., 2017). Although these plasmids come from different host strains, and even from different species in different geographic locations, the genome comparison of the plasmid sequences showed that they are highly similar, which means that most of them have spread in various *Enterobacteriaceae* worldwide.

We conducted conjugation experiments on the eight *mcr-1* positive nontyphoidal *Salmonella* strains, and found that all eight strains were successfully conjugated, indicating that all of the *mcr-1* genes were located on the plasmid. The results of S1-PFGE and Southern blotting showed that seven of the eight strains of plasmids carrying *mcr-1* belonged to IncHI2 with approximately 220–280kb, which is consistent with the recently reported plasmid carrying *mcr-1* of ST34 *S.* Typhimurium in Zhejiang, China. Notedly, the *S.* Sinstorf serotype strain belongs to the IncI2-type plasmid with 60kb, which has not been reported before. All of these results indicate that the *mcr-1* gene plays an important role in the colistin resistance of clinically isolated nontyphoidal *Salmonella* strains and its spread can lead to a rapid increase in colistin resistance.

In recent years, due to the unreasonable clinical and agricultural use of antibiotics, the antimicrobial resistance rate of *Salmonella* has increased rapidly. According to previous reports, *mcr-1*-positive isolates are usually still susceptible to many other antibiotics (Eiamphungporn et al., 2018). In our study, eight strains of *mcr-1* positive strains were resistant to sulfisoxazole, and 87.5% (7/8) and 75% (6/8) showed resistance to third-generation cephalosporins and ciprofloxacin, respectively. It is worth noting that in our study, five colistin-intermediate resistance nontyphoidal *Salmonella* with MIC=2 were also found to carry the *mcr-1* gene. Due to the existence of the *mcr-1* gene, *Salmonella* is in the process of transforming from being susceptible to colistin to resistant. Moreover, difference may also be due to their level of expression on the different plasmids or in the different genomic backgrounds. Sequencing analysis showed that 62.5% (5/8) of these isolates were *bla*_CTX-M-14_ ESBL-producing strains, and these isolates also harbored quinolone resistance genes *qnrS* and/or *aac(6′)-Ib-cr4*, and their genotypes were consistent with their resistant phenotypes. Therefore, comprehensive monitoring of *mcr-1* carrying status and plasmid structure in clinical *Salmonella* isolates within Jiangsu and understanding the transmission characteristics and mechanism of *mcr-1* in nontyphoidal *Salmonella* may provide a preliminary basis for controlling the spread of colistin resistance among Gram-negative bacterial pathogens in the future.

## Conclusion

In summary, we reported the prevalence and resistance mechanism of the occurrence of nontyphoidal *Salmonella* isolates in Jiangsu, China. We found that resistance to ciprofloxacin, cephalosporins, and colistin were wide-spread. We identified the related mutations and various transferrable antimicrobial-resistance genes, which included ESBL, PMQR and *mcr-1*, and some isolates harbored two or more types of these genes. The dissemination of these genes poses a huge threat to the control of nontyphoidal *Salmonella* infection around the world. Additionally, the present study reported the prevalence of the *mcr-1* gene among nontyphoidal *Salmonella* in Jiangsu, China. Notably, diverse STs of *mcr-1* harboring nontyphoidal *Salmonella* were mainly located on ∼220–280kb IncHI2 plasmids, indicating that the fast evolution and high transfer ability of this kind of plasmid has led to the high prevalence of the *mcr-1* gene. Interestingly, one *Salmonella* strain harboring *mcr-1* gene with a new ST type ST155 and new *enterica* serovar Sinstorf has never been detected before in human. Moreover, the *mcr-1* belonged to an IncI2-type plasmid of 60kb. Hence, timely detection of the *mcr-1* gene and antimicrobial susceptibility testing are necessary so that infections caused by *mcr-1* carriers receive appropriate and effective therapy. Similarly, large-scale surveillance and effective infection control measures are also urgently needed to prevent the spread of *mcr-1* carriers.

## Data Availability Statement

The raw data supporting the conclusions of this article will be made available by the authors, without undue reservation.

## Author Contributions

GL, HY, and BG designed the hypothesis. CB collected and integrated strain information. GL and HQ performed the experimental work. JL analyzed the computationally generated data. GL and BT prepared the draft manuscript. HY and BG commented on the final manuscript. All authors contributed to the article and approved the submitted version.

## Conflict of Interest

The authors declare that the research was conducted in the absence of any commercial or financial relationships that could be construed as a potential conflict of interest.

## Publisher’s Note

All claims expressed in this article are solely those of the authors and do not necessarily represent those of their affiliated organizations, or those of the publisher, the editors and the reviewers. Any product that may be evaluated in this article, or claim that may be made by its manufacturer, is not guaranteed or endorsed by the publisher.

## Footnotes

^1^ http://www.genomicepidemiology.org/
