# Supplementary material for: Emergence of mcr-1-Harboring Salmonella enterica Serovar Sinstorf Type ST155 Isolated From Patients With Diarrhea in Jiangsu, China
Source: Front Microbiol. 2021 Sep 3;12:723697. doi: 10.3389/fmicb.2021.723697 (PMC8483771; doi:10.3389/fmicb.2021.723697)
Supplement: Supplementary file 1 [file Table_1.DOCX]

**Table S1 Distribution of PMQR genes and mutations in gyrA, gyrB, parC, and parE genes in NTS isolates in Jiangsu, China.**

| CIP | | | QRDR mutation | | | | PMQR | Total |
| --- | --- | --- | --- | --- | --- | --- | --- | --- |
| S | I | R | *GyrA* | *GyrB* | *ParC* | *ParE* |  |  |
| 1 | 0 | 0 | WT | WT | WT | WT | qnrA1 | 1 |
| 0 | 0 | 1 | WT | WT | WT | WT | aac(6’)-ib-cr4, qnrA1 | 1 |
| 0 | 0 | 1 | WT | WT | WT | WT | qnrS1, aac(6’)-ib-cr4,qnrB6 | 1 |
| 13 | 37 | 16 | WT | WT | WT | WT | qnrS1 | 66 |
| 0 | 1 | 0 | WT | WT | WT | WT | qnrS1, aac(6’)-ib-cr4 | 1 |
| 0 | 0 | 1 | WT | WT | WT | WT | qnrS1, qnrS2 | 1 |
| 0 | 0 | 1 | WT | WT | WT | WT | qnrS1, qnrS2, aac(6’)-ib-cr4 | 1 |
| 0 | 0 | 1 | WT | WT | WT | WT | qnrS2, aac(6’)-ib-cr4 | 1 |
| 0 | 0 | 2 | WT | WT | WT | WT | qnrS2 | 2 |
| 1 | 5 | 5 | WT | WT | WT | WT | qnrB4 | 11 |
| 0 | 0 | 2 | WT | WT | WT | WT | qnrB4, aac(6’)-ib-cr4 | 1 |
| 3 | 2 | 1 | WT | WT | WT | WT | qnrB6 | 6 |
| 1 | 4 | 7 | WT | WT | WT | WT | qnrB6, aac(6’)-ib-cr4 | 12 |
| 14 | 3 | 11 | WT | WT | WT | WT | aac(6’)-ib-cr4 | 28 |
| 0 | 0 | 1 | S83A | WT | T57S | L440R, R508K, N516H, A545E | qnrS2 | 1 |
| 0 | 0 | 1 | S83A | WT | WT | WT | WT | 1 |
| 0 | 3 | 0 | S83F | WT | WT | WT | WT | 3 |
| 0 | 3 | 0 | S83F | WT | T57S | WT | WT | 3 |
| 0 | 1 | 0 | S83F | WT | T57S | WT | aac(6’)-ib-cr4 | 1 |
| 0 | 0 | 3 | S83F/D87G | WT | T57S/S80I | WT | WT | 3 |
| 0 | 0 | 1 | S83F/D87G | WT | T57S | WT | WT | 1 |
| 0 | 0 | 1 | S83F/D87N | WT | T57S/S80I | WT | WT | 1 |
| 0 | 0 | 1 | S83F/D87N | WT | T57S/S80R | WT | aac(6’)-ib-cr4 | 1 |
| 0 | 0 | 1 | S83F/D87N | WT | T57S/S80I | WT | qnrS1 | 1 |
| 0 | 1 | 0 | S83F/D87Y | WT | WT | WT | WT | 1 |
| 0 | 1 | 0 | S83F/E133G | WT | WT | WT | WT | 1 |
| 0 | 0 | 1 | S83L/D87G/K154R | WT | WT | A512T/N516E/A545T | qnrS1, aac(6’)-ib-cr4 | 1 |
| 0 | 0 | 1 | S83L | WT | WT | D473N/R508K/N516H/ A545E | WT | 1 |
| 0 | 1 | 0 | S83L | WT | T57S | WT | WT | 1 |
| 0 | 0 | 1 | S83L/D87N | WT | WT | WT | WT | 1 |
| 0 | 0 | 1 | S83L/D87G | WT | WT | R508K/N516H/ A545E | qnrS1 | 1 |
| 0 | 0 | 1 | S83T/I112V/L127M/A128S/K154R | WT | T57S | Y434F/R508K/A512T/N516H | WT | 1 |
| 0 | 1 | 0 | S83T/I112V/L127M/A128S/K154R | WT | T57S | R508K/A512T/N516H | qnrD1 | 1 |
| 0 | 1 | 0 | S83T/I112V/L127M/A128S/K154R | WT | WT | R508K/A512T/N516H | WT | 1 |
| 0 | 19 | 1 | S83Y | WT | WT | WT | WT | 20 |
| 0 | 5 | 0 | S83Y | WT | T57S | WT | WT | 5 |
| 0 | 1 | 0 | S83Y | WT | T57S/S80I | WT | WT | 1 |
| 0 | 0 | 2 | S83Y | WT | WT | WT | qnrS1 | 2 |
| 1 | 28 | 1 | D87G | WT | WT | WT | WT | 30 |
| 0 | 0 | 1 | D87G | WT | WT | WT | qnrS1 | 1 |
| 0 | 1 | 0 | D87G | WT | WT | WT | aac(6’)-ib-cr4 | 1 |
| 0 | 4 | 9 | D87N | WT | WT | WT | aac(6’)-ib-cr4 | 13 |
| 0 | 0 | 1 | D87N | WT | WT | WT | qnrS1 | 1 |
| 1 | 9 | 8 | D87N | WT | WT | WT | WT | 18 |
| 1 | 76 | 5 | D87Y | WT | WT | WT | WT | 82 |
| 0 | 3 | 0 | D87Y | WT | T57S | WT | WT | 3 |
| 0 | 4 | 1 | D87Y | WT | WT | WT | aac(6’)-ib-cr4 | 5 |
| 0 | 1 | 0 | D87Y | WT | WT | WT | qnrS1 | 3 |
| 0 | 3 | 0 | D87Y | WT | WT | WT | qnrS1, aac(6’)-ib-cr4 | 1 |
| 0 | 0 | 1 | E133G | WT | WT | WT | WT | 1 |
| 0 | 2 | 2 | WT | WT | T57S | WT | qnrS1 | 4 |
| 0 | 0 | 1 | WT | WT | T57S | WT | qnrS2 | 1 |
| 0 | 0 | 1 | WT | WT | T57S | WT | qnrS2, aac(6’)-ib-cr4 | 1 |
| 0 | 1 | 0 | WT | WT | T57S | WT | qnrB19 | 1 |
| 0 | 0 | 2 | WT | WT | T57S | WT | qnrB6, aac(6’)-ib-cr4 | 2 |
| 0 | 1 | 2 | WT | WT | T57S | WT | aac(6’)-ib-cr4 | 3 |
| 0 | 1 | 0 | WT | WT | T57S | WT | qnrD1 | 1 |
| 1 | 4 | 3 | WT | WT | T57S | WT | WT | 8 |

**Table S2 Summary of phenotypes of NTS isolates showing concurrently resistance to ciprofloxacin and cefotaxime and their corresponding resistance genes in 2016.**

| Sample ID | Serotype | Year | City | MIC (mg/liter) | |  | QRDR amino acid substitutionsa | | |  | Plasmid-mediated resistance | |
| --- | --- | --- | --- | --- | --- | --- | --- | --- | --- | --- | --- | --- |
|  |  |  |  | CTX | CIP |  | *GyrA* | *ParC* | *ParE* |  | PMQR | *β-*lactams |
| SA16005 | S.Typhimurium | 2016 | Huai'an | ＞8 | 2 |  | D87N | / | / |  | *aac(6’)-ib-cr4* | *TEM-1* |
| SA16014 | S.Indiana | 2016 | Huai'an | ＞8 | 4 |  | / | / | / |  | */* | *TEM-1, CTX-M-14* |
| SA16029 | S.Typhimurium | 2016 | Huai'an | ＞8 | 16 |  | S83F/D87N | T57S/S80R | / |  | *aac(6’)-ib-cr4* | *TEM-1, CTX-M-65* |
| SA16042 | S.Typhimurium | 2016 | Lianyungang | ＞8 | 16 |  | / | / | / |  | */* | *TEM-1, mcr-1* |
| SA16050 | S.Lille | 2016 | Lianyungang | ＞8 | 8 |  | / | / | / |  | */* | *TEM-1* |
| SA16074 | S.Thompson | 2016 | Nanjing | ＞8 | 16 |  | / | / | / |  | */* | *TEM-1* |
| SA16080 | S.Typhimurium | 2016 | Nanjing | ＞8 | 8 |  | / | / | / |  | */* | *TEM-1* |
| SA16082 | S.Typhimurium | 2016 | Nanjing | ＞8 | 32 |  | / | / | / |  | */* | *TEM-1, CTX-M-14* |
| SA16083 | S.Enteritidis | 2016 | Nanjing | ＞8 | 1 |  | D87Y/ E133G | / | / |  | */* | *TEM-1, CTX-M-14* |
| SA16084 | S.Enteritidis | 2016 | Nanjing | 8 | 8 |  | / | / | / |  | */* | *TEM-1* |
| SA16090 | S.Farsta | 2016 | Nanjing | ＞8 | 16 |  | / | / | / |  | */* | *TEM-1* |
| SA16096 | S.London | 2016 | Nanjing | ＞8 | 1 |  | / | / | / |  | *aac(6’)-ib-cr4* | *TEM-1, CTX-M-14* |
| SA16103 | S.Thompson | 2016 | Nanjing | ＞8 | 16 |  | / | / | / |  | */* | *TEM-1* |
| SA16128 | S.Aberdeen | 2016 | Nantong | ＞8 | 4 |  | / | / | / |  | */* | *TEM-1, CTX-M-14, CMY-2* |
| SA16142 | S.Rissen | 2016 | Nantong | ＞8 | 1 |  | / | / | / |  | *aac(6’)-ib-cr4* | *TEM-1* |
| SA16144 | S.Typhimurium | 2016 | Nantong | ＞8 | 4 |  | / | / | / |  | */* | *TEM-1, CMY-2* |
| SA16158 | S.Typhimurium | 2016 | Suzhou | ＞8 | 1 |  | / | / | / |  | */* | *TEM-1, CTX-M-65* |
| SA16163 | S.Typhimurium | 2016 | Suzhou | ＞8 | 1 |  | D87N | T57S/S80I | / |  | */* | *TEM-1, CTX-M-14* |
| SA16176 | S.Fillmore | 2016 | Suzhou | 4 | 2 |  | / | / | / |  | *aac(6’)-ib-cr4* | *TEM-1* |
| SA16205 | S.Seremban | 2016 | Taizhou | ＞8 | 2 |  | / | / | / |  | *aac(6’)-ib-cr4* | *TEM-1* |
| SA16210 | S.Typhimurium | 2016 | Taizhou | ＞8 | 2 |  | / | / | / |  | *aac(6’)-ib-cr4* | *TEM-1* |
| SA16213 | S.Lagos | 2016 | Taizhou | 8 | 2 |  | / | / | / |  | *aac(6’)-ib-cr4* | *TEM-1* |
| SA16214 | S.Lagos | 2016 | Taizhou | ＞8 | 4 |  | / | / | / |  | *aac(6’)-ib-cr4* | *TEM-1, CTX-M-14, mcr-1* |
| SA16237 | S.Typhimurium | 2016 | Suqian | ＞8 | 8 |  | / | / | / |  | *qnrS2* | *TEM-1, mcr-1* |
| SA16251 | S.Typhimurium | 2016 | Suqian | ＞8 | 1 |  | / | / | / |  | *qnrS1* | *TEM-1, CTX-M-65* |
| SA16257 | S.Thompson | 2016 | Suqian | ＞8 | 16 |  | / | / | / |  | *qnrS1* | *TEM-1, CMY-2* |
| SA16262 | S.Typhimurium | 2016 | Xuzhou | ＞8 | 1 |  | / | / | / |  | *qnrS1* | *TEM-1* |
| SA16264 | S.Bonariensis | 2016 | Xuzhou | ＞8 | 16 |  | S83F/D87G | / | / |  | */* | *TEM-1, CTX-M-14* |
| SA16277 | S.Typhimurium | 2016 | Xuzhou | ＞8 | 1 |  | D87N | / | / |  | *aac(6’)-ib-cr4* | *TEM-1, CTX-M-14* |
| SA16280 | S.Braenderup | 2016 | Xuzhou | ＞8 | 8 |  | / | / | / |  | *qnrS1* | *TEM-1, CMY-2* |
| SA16281 | S.Typhimurium | 2016 | Xuzhou | ＞8 | 8 |  | / | / | / |  | *qnrS1* | *TEM-1* |
| SA16289 | S.Bonariensis | 2016 | Xuzhou | ＞8 | 16 |  | S83F/D87G | T57S/S80I | / |  | */* | *TEM-1, CTX-M-14* |
| SA16305 | S.Thompson | 2016 | Yancheng | ＞8 | 8 |  | / | / | / |  | *qnrS1* | *TEM-1, CMY-2* |
| SA16307 | S.Choleraesuis | 2016 | Yancheng | ＞8 | 8 |  | D87G | / | / |  | *qnrS1* | *TEM-1, CMY-2* |
| SA16308 | S.Choleraesuis | 2016 | Yancheng | ＞8 | 2 |  | D87N | / | / |  | *aac(6’)-ib-cr4* | *TEM-1, CTX-M-14* |
| SA16314 | S.Montevideo | 2016 | Zhenjiang | ＞8 | 1 |  | / | / | / |  | *qnrB4* | *TEM-1* |
| SA16316 | S.Stanley | 2016 | Zhenjiang | 8 | 2 |  | / | / | / |  | *qnrB4* | *TEM-1* |
| SA16341 | S.Kentucky | 2016 | Zhenjiang | ＞8 | 16 |  | / | / | / |  | */* | *TEM-1* |
